# In Vitro Cross-Linking MS Reveals SMG1–UPF2–SMG7 Assembly as Molecular Partners within the NMD Surveillance

**DOI:** 10.3390/ijms25063182

**Published:** 2024-03-10

**Authors:** Monikaben Padariya, Borivoj Vojtesek, Ted Hupp, Umesh Kalathiya

**Affiliations:** 1 International Centre for Cancer Vaccine Science, University of Gdansk, ul. Kładki 24, 80-822 Gdansk, Poland; ted.hupp@ed.ac.uk; 2 Research Centre for Applied Molecular Oncology, Masaryk Memorial Cancer Institute, Zluty kopec 7, 656 53 Brno, Czech Republic; vojtesek@mou.cz; 3 Edinburgh Cancer Research Centre, Institute of Genetics and Molecular Medicine, University of Edinburgh, Edinburgh EH4 2XR, UK

**Keywords:** NMD, mRNA, cross-linking, mass spectrometry, UPF2, SMG1, SMG7, cancer mutations, protein stability

## Abstract

mRNAs containing premature stop codons are responsible for various genetic diseases as well as cancers. The truncated proteins synthesized from these aberrant mRNAs are seldom detected due to the nonsense-mediated mRNA decay (NMD) pathway. Such a surveillance mechanism detects most of these aberrant mRNAs and rapidly destroys them from the pool of mRNAs. Here, we implemented chemical cross-linking mass spectrometry (CLMS) techniques to trace novel biology consisting of protein–protein interactions (PPIs) within the NMD machinery. A set of novel complex networks between UPF2 (Regulator of nonsense transcripts 2), SMG1 (Serine/threonine-protein kinase SMG1), and SMG7 from the NMD pathway were identified, among which UPF2 was found as a connection bridge between SMG1 and SMG7. The UPF2 N-terminal formed most interactions with SMG7, and a set of residues emerged from the MIF4G-I, II, and III domains docked with SMG1 or SMG7. SMG1 mediated interactions with initial residues of UPF2, whereas SMG7 formed very few interactions in this region. Modelled structures highlighted that PPIs for UPF2 and SMG1 emerged from the well-defined secondary structures, whereas SMG7 appeared from the connecting loops. Comparing the influence of cancer-derived mutations over different CLMS sites revealed that variants in the PPIs for UPF2 or SMG1 have significant structural stability effects. Our data highlights the protein–protein interface of the SMG1, UPF2, and SMG7 genes that can be used for potential therapeutic approaches. Blocking the NMD pathway could enhance the production of neoantigens or internal cancer vaccines, which could provide a platform to design potential peptide-based vaccines.

## 1. Introduction

A variety of pathways within cells control the quality as well as quantity of mRNA beyond the end points of transcription [1]. Among them, nonsense-mediated mRNA decay (NMD) is an important cellular surveillance mechanism that identifies and degrades aberrant mRNA (containing premature termination codons; PTCs). These PTCs in mRNA are caused by frameshift mutations, pseudogenes, or alternative splicing events [2], which if translated into proteins cause negative inhibitory effects. PTC transcripts that are more than 50 nucleotides upstream of the last exon–exon junction (EJC) are degraded by cytoplasmic and translation-dependent NMD. Despite stop codons at the end of normal transcripts preventing EJCs upon translation termination of mRNA, PTCs delay the ribosome and recruit NMD-related proteins to degrade aberrant mRNA [3,4]. There is an estimation that 30% of mutations result in PTCs in mRNA, which are responsible for human genetic disorders [5].

Though the molecular mechanism of NMD machinery is still unclear, a working model of how NMD breaks down mRNA containing PTCs is postulated. Upon paused mRNA translation due to mRNA with PTCs in the upstream of EJC, the eukaryotic release factors (eRFs) recruit the master regulator of the NMD pathway: the UPF1 mRNA helicase [6]. eRF1 protein could identify all three UAG, UGA, and UAA stop codons [7] in mRNA transcripts. Particularly, for the UPF1 gene, phosphorylation and dephosphorylation are crucial steps before the degradation of the transcript by exonucleases [8]. Along with the master regulator (UPF1; Up-frameshift 1), the NMD pathway is a multiprotein complex that consists of the hUPFs (UPF2; Regulator of nonsense transcripts 2, UPF3a, and UPF3b), the suppressors with morphological effects on genitalia proteins (SMG1; Serine/threonine-protein kinase SMG1, SMG5 –SMG9), and the EJC (EIF4A3; eukaryotic translation initiation factor 4A3, MAGOH; mago homolog, RBM8A; RNA-binding motif protein 8A, and Barentsz; BTZ) [9].

The inter-relational functionality of NMD components gives directions about the conserved protein–protein interfaces or interactions. Upon activation of the NMD process, it initiates the formation of the SURF surveillance network consisting of UPF1, SMG1, eRF1, and eRF3 genes. This SURF complex interacts with the UPF2, UPF3b, and an EJC downstream of the PTC, forming the decay-inducing complex (DECID) [10]. In particular, the UPF3b docks with the EJC along with anchoring with the UPF2 protein. The phosphorylation of UPF1 by SMG1 is promoted by UPFs and the SURF complex, whereas the dephosphorylation is a multiprotein process (SMG5, SMG6, SMG7, and PP2A; protein phosphatase 2A) [11]. Recruitment of SMG7 to UPF1 targets the mRNA containing PTCs for degradation and interactions of PP2A with SMG7 dephosphorylate UPF1 and prepares a second NMD round. Allowing for the fine-tuning of the NMD activity, the UPF3a protein inhibits NMD, and this activity is regulated by the UPF3b protein [12]. It has been reported that RNA binding and ATPase/unwinding activity of the UPF1 gene is regulated by UPF2. The UPF1 RNA-unwinding activity is increased upon interacting with UPF2, the UPF1 CH-domain is displaced, and domain 1B changes to a conformation where it does not clamp on the mRNA 3′ end [13,14].

It has been suggested that NMD inhibition can be achieved via other mechanisms and determined that a modest 80% depletion of UPF1 can suppress NMD activity without diminishing the proliferation or survival of cells [8]. Deletion of UPF1 hugely increases the production of novel peptide readthrough, which is a massively important advance in the need to block the NMD process to stimulate cancer vaccines [15]. There are small molecules, such as aminoglycosides (oto- and nephrotoxic), dipeptides, and oxadiazoles (Ataluren) [16] that promote the “read-through” or “suppression” of nonsense mutations. NMD inhibitors can be combined with drugs that promote translation readthrough at PTCs (e.g., PTC124 and aminoglycosides such as G418 and gentamicin) to produce full-length protein products [17,18]. As a proof of concept, co-treatment with the NMD inhibitors NMDI14 and G418 restored full-length p53, causing subsequent cell death in cells expressing nonsense p53 mRNA [8]. Although previous studies identified NMD inhibitors with different mechanisms of action (NMDI-1 disrupts the interaction of the NMD factors UPF1-SMG5; NMDI-14 disrupts the UPF1–SMG7 interaction [8,19]) there is still a need and a place for new therapeutic strategies.

Understanding the interacting partners or the components from the NMD, EJC, and eRFs as well as their involvement in different biological pathways suggests that they are involved in several critical and important functions. Along with the NMD process, the multiprotein complexes, or components from the NMD, EJC, and eRF complexes are widely involved in the regulation of telomere maintenance, regulation of chromosome organization, and RNA transport/localization/catabolic processes (Figure S1 in the Supporting Materials). Looking over the mutational landscape of NMD, EJC, and eRF components from different cancer types suggests that these proteins are heavily mutated in several cancers. Therefore, this suggests that a detailed understanding of the protein–protein network is crucial and investigating the mutational effect on the stability and binding of protein–protein can provide new insights into the molecular mechanisms of this complex process and assist in the development of novel therapeutic approaches. Implementing a cross-linking mass spectrometry (CLMS [20,21]) approach and using the non-cleavable DSS (disuccinimidyl suberate) cross-linker, we identified novel interaction sites within the NMD pathway components SMG1–UPF2–SMG7. The DSS cross-linker, with a spacer arm length of 11.4 Å, undergoes covalent bond formation targeting Lysine (K) or Serine (S) residues [20,22]. In our previous study [21], we validated crucial interacting proteins by applying a similar method by co-immunoprecipitation (Co-IP), preferentially often using a cross-linker range of 10–12.5 Å space length for the cleavable (DSS, BS3, etc.) and non-cleavable (DSSO, DSBU, etc.) cross-linkers. Such CLMS can even be performed with shorter-length spacer arm cross-linkers, for example, 8.8 Å (PAC4), 5 Å (PhoX), etc. [20]. Our CLMS data with an optimal DSS cross-linker (avoiding over-cross-linking complexes) highlight interaction between protein–protein interfaces (PPIs) of SMG1, UPF2, and SMG7 proteins that can be used for potential therapeutic approaches.

## 2. Results and Discussions

### 2.1. Inter- and Intramolecular Interactions between UPF2, SMG1, and SMG7

**Figure 1:**
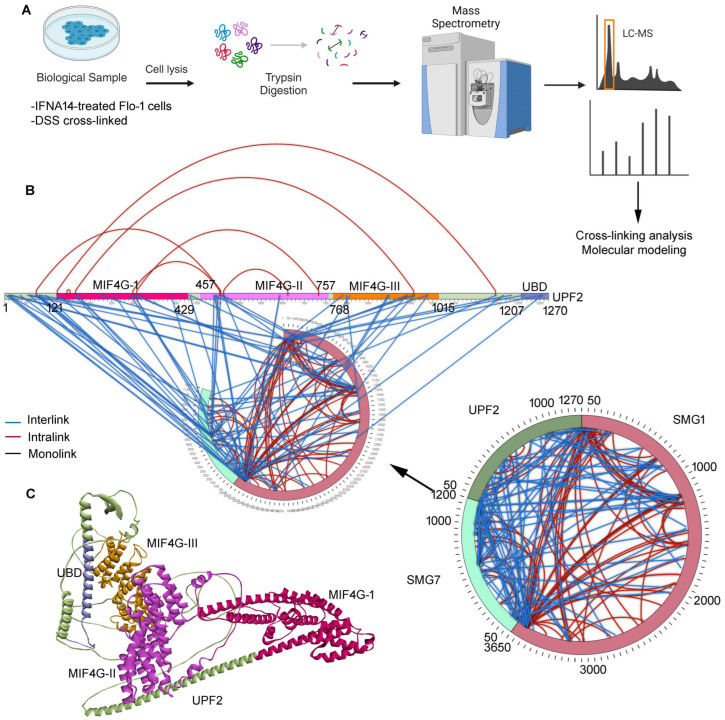
Identification of SMG1–UPF2–SMG7 interactions through chemical cross-linking mass spectrometry (CLMS) approach. (**A**) Brief outline of protocol implemented to identify protein–protein networks [21] (www.biorender.com). As described in our previous work [21], the Flo-1 cancer cells were treated with DSS cross-linker and IFNa14 interferon to identify upregulated protein–protein interactions (PPIs). (**B**) Identified SMG1–UPF2–SMG7 complex using CLMS technique. The blue line represents intermolecular interactions and red describes the intramolecular network. The UPF2 protein network is represented in 2D format, and the right panel shows circular representations of SMG1–UPF2–SMG7 interactions. (**C**) Modelled full-length structure of UPF2 genes from the NMD pathway, individual domains are highlighted in different colours (MIF4GI-III; middle of 4G-like domains; UBD; UPF1-binding domain).

Chemical cross-linking combined with mass spectrometry has been widely used to analyze protein structures and protein–protein interactions in a qualitative manner. The Flo-1 cancer cells treated with IFNα14 and cross-linked using the optimal DSS cross-linker revealed several interactions [1], among which we identified a set of novel complex networks between UPF2, SMG1, and SMG7 (Figure 1) from the NMD pathway [23,24].

Along with having a crucial role in identifying the SMG1–UPF2–SMG7 complex within the NMD process, the SMG1 genes have been shown distinguishable in tasks in the alternative splicing of the tumour-suppressor p53 genes [25]. Due to the different activities of SMG1 in multiple myeloma (MM) cell lines [26], it has become a promising therapeutic target for the development of novel drug molecules. The activation of the SMG1-UPF within the NMD pathway has shown a downregulation of negative regulator MDM2 (Murine Double Minute Clone 2) by Glaucocalyxin A in Gastric cancer (GC) cells [27]. Knockouts of SMG7 in different cancer cell lines have been shown to reduce or suppress the NMD activity [28] and disrupting SMG7 function may provide therapeutic benefits [29]. DNA damage enhances the binding of SMG7-p53, which requires Ser15 phosphorylation of p53. In addition, mutation of K66M in SMG7 blocks the binding of p53 and UPF1 [30]. It has been reported that SMG7 has an important role in the p53-mediated response to genotoxic stress by regulating p53 stability, and SMG7 physically interacts with MDM2 [31].

Independent biological replicates of experiments have confirmed interactions between SMG1, UPF2, and SMG7 (Figure S2), as well as suggesting that UPF2 can play a central role in interconnecting SMG1 and SMG7 within the NMD machinery. Specifically, the CLMS data revealed that UPF2 has a set of binding sites interacting with SMG1 or SMG7 (Figure 1B). The UPF2 N-terminal formed several interactions with SMG7, and a set of residues emerged from the MIF4G-I to III (middle of 4G-like domains) domains docked with SMG1 or SMG7. However, the UPF2 MIF4G-II domain defined most of the interactions with SMG7 (Figure 1B). In addition, the UPF2 MIF4G-I domain formed intramolecular interactions with the MIF4G-II and III domains (Figure 1B). Comparing these interactions with the homology-modelled structure of the UPF2 protein revealed that most of them emerged from the well-defined helical structures (Figure 1C).

**Figure 2:**
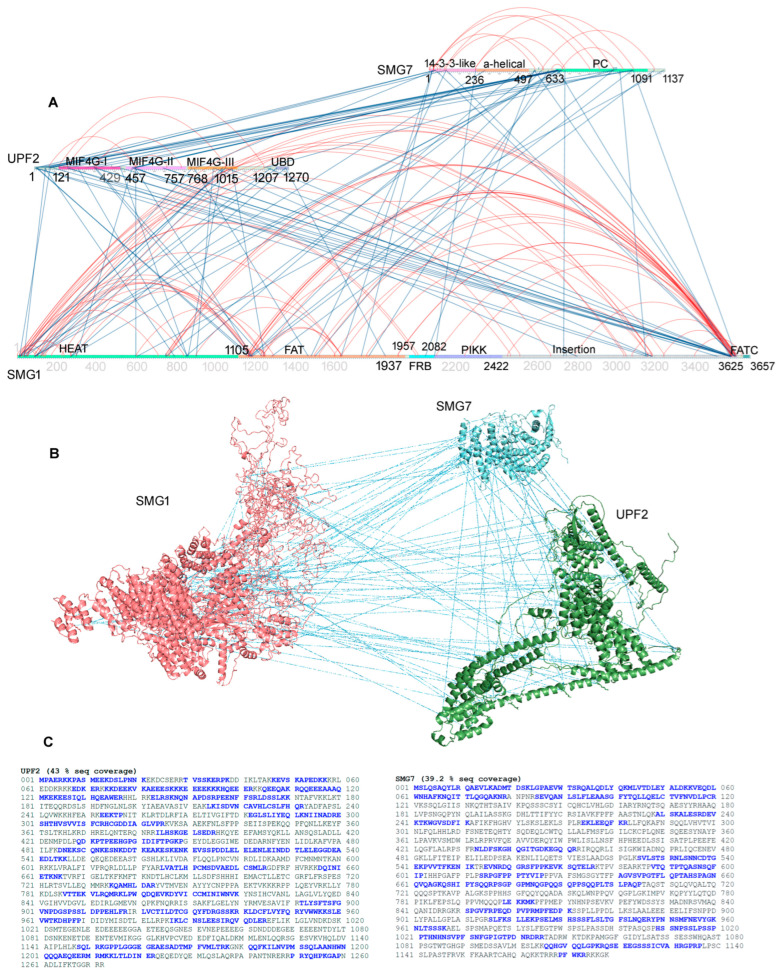
The domain-wise distribution of protein–protein interactions (PPIs) between the SMG1–UPF2–SMG7 complex within the NMD machinery. (**A**) Interactions plot of SMG1, UPF2, and SMG7 from the NMD pathway. Inter- and intramolecular interactions are presented in blue and red, respectively. (**B**) Full-length structures of SMG1, UPF2, and SMG7 genes and their intermolecular interactions identified in cross-linking MS experiments are shown as blue lines. (**C**) Sequence coverage of UPF2 and SMG7 genes based on the cross-linked peptides identified that are involved in the PPIs. Blue sequences are highlighted showing intermolecular interactions. The SMG1 domains, HEAT (Huntingtin), FAT (focal adhesion kinase domain), PIKK (phosphatidylinositol 3-kinase-related protein kinase domain), FATC (C-terminal FAT domain), SMG7 domains, and PC (C-terminal proline-rich region) are highlighted by different colours.

Along with different PPIs, the cross-linking MS approach identified several intramolecular interactions within the SMG1, UPF2, or SMG7 (Figure 2A and Table S1). The SMG1 C-terminal region (3552 to 3558 amino acids; aa), near the FATC (C-terminal FAT) domain, formed interactions with the HEAT (Huntingtin; 1–148 aa) and FAT (focal adhesion kinase domain; 1109–1156 aa) regions. Comparing the full-length modelled structure of SMG1 (Figure 2B) and such intramolecular interactions between different domains, it can be postulated there could be a conformational switch of the SMG1 structure when networking with different proteins (Figure 2B). For the SMG7 gene, the 14-3-3-like domain has shown intramolecular binding with the PC (C-terminal proline-rich region) domain (Figure 2A).

Analyzing the intermolecular interactions between the SMG1–UPF2–SMG7 complex, it was observed that SMG1 C-terminal (3550–3600 aa) mainly interacts with the UPF2 N-terminal (10–25 aa), MIF4G-I (160–170 and 300–310 aa), and MIF4G-II (490–510) domains, whereas the SMG1 N-terminal binds with MIF4G-III (940–1000 aa; Figure 2A). Subsequently, the UPF2 N-terminal (10–90 aa) has formed interactions with regions nearby to the PC (630–660 aa) of the SMG7 gene (Figure 2B). A lack of consistent interaction spots between SMG1–SMG7 complexes was revealed in our cross-linking analysis, and their PPI sites were scattered over the entire sequences representing multiple structure conformations (Figure 2). Therefore, our findings suggest that UPF2 can be the component connecting both SMG1 and SMG7 proteins within the NMD process, playing a crucial role in initializing the mRNA degradation process with UPF1. Implementing homology modelling approaches, we constructed full-length structures of all three cross-linked genes and overlayed the identified cross-linked MS interactions on their structures, which revealed in many cases the specific hotspots belonging to the well-defined regions (Figure 2B). Analyzing the sequence coverage of cross-linked peptides identified within the Flo-1 cells treated with DSS cross-linker suggests several specific sites interacting with these SMG1–UPF2–SMG7 proteins (Figure 2C and Figure S3).

**Figure 3:**
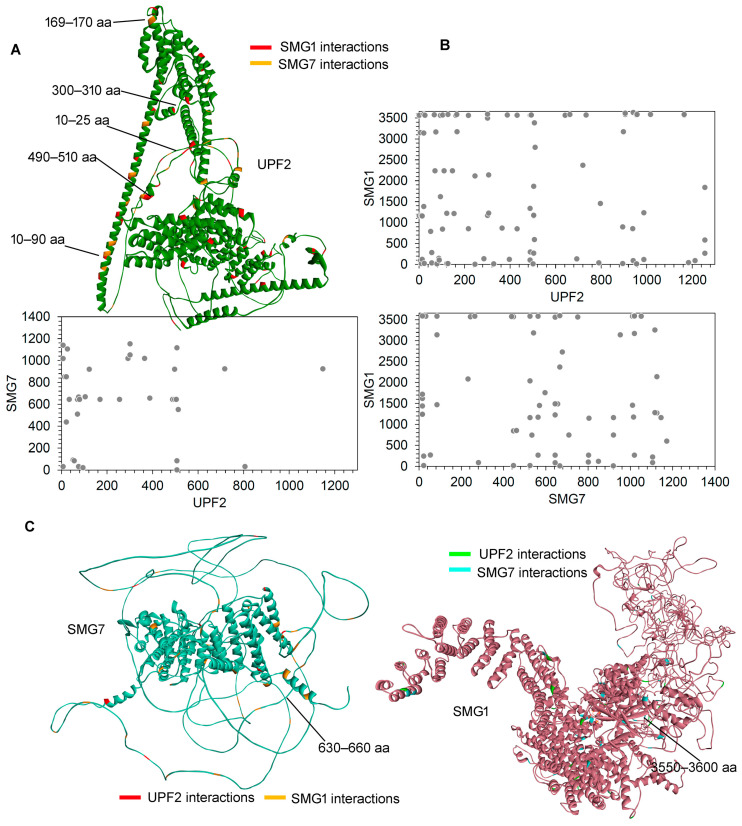
Layout of cross-linking MS-derived interactions of the protein structure from the SMG1–UPF2–SMG7 complex. (**A**) Interactions of UPF2 with SMG1 and SMG7 were identified using cross-linking MS technique. The interactions between UPF2-SMG1 and UPF2-SMG7 are marked in red and orange, respectively. (**B**) Interactions plots of SMG1, UPF2, SMG7 networks. (**C**) The network of SMG7 with UPF2 and SMG1. The interactions between UPF2-SMG7 are highlighted in red and SMG1–SMG7 are marked in orange colour. The right panel represents SMG1 with UPF2 and/or SMG7. The interactions between UPF2-SMG1 are highlighted in green and SMG1–SMG7 are marked in blue colour.

Overlaying the cross-linked identified interactions in Flo-1 cancer cells revealed that SMG1 and SMG7 formed different spots (suggesting multiple conformations captured using CLMS) of interactions with UPF2 in many cases (Figure 3A). SMG1 was found to have extensive interactions with UPF2 mostly with the initial residues, whereas SMG7 formed very few specific interactions in this region (Figure 3B). The SMG7 residues within the range 630–660 aa formed interactions with UPF2 that emerged from the connecting loop region (Figure 3C). The SMG1 gene, where initial residues formed interactions with UPF2, later was found to bind with SMG7 (Figure 3). Modelling full-length structures of SMG1, UPF2, and SMG7, it was observed that for UPF2 and SMG1 interactions emerged from the well-defined secondary structures, whereas for SMG7 appeared from the connecting loops (Figure 3); however, visualizing the model structures of SMG1, UPF2, and SMG7, we propose that a more accurate method such as structural optimization could be implemented using the molecular dynamic simulation (MDS) approach (Figure 3C).

**Figure 4:**
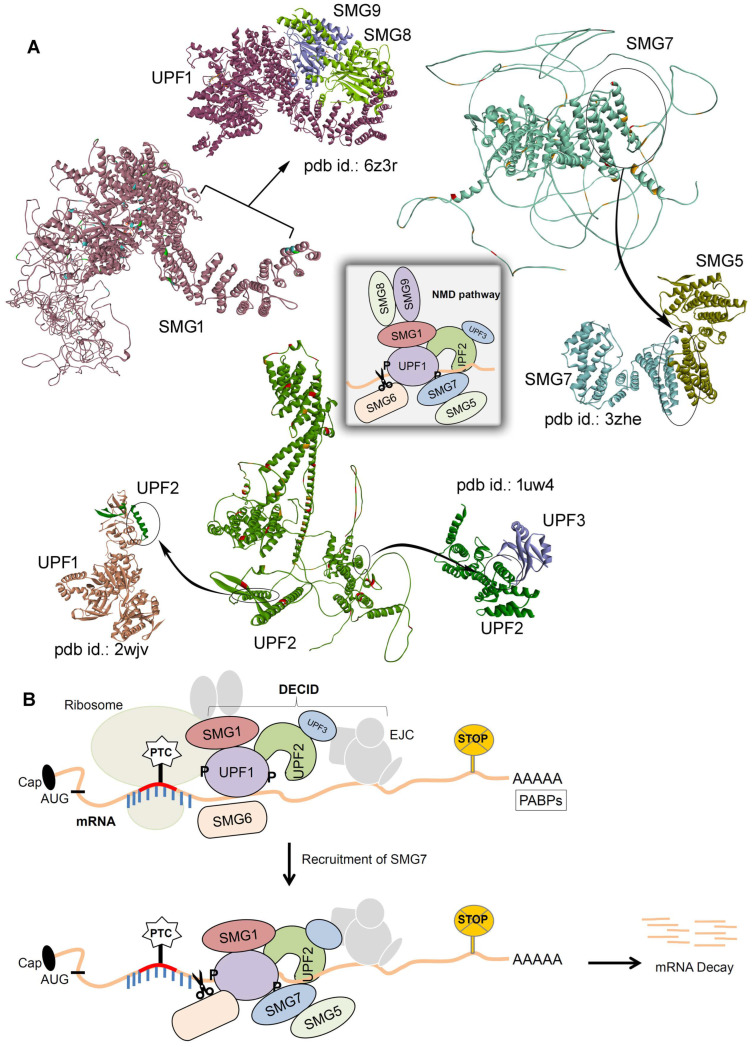
Proposed mechanism of the identified SMG1–UPF2–SMG7 PPIs. (**A**) Investigating existing structures of SMG1, UPF2, and SMG7 genes revealed that they play a crucial role in activating the NMD process, which results in the degradation of the premature termination stop codon containing mRNAs. The following known structures can be found in the rcsb pdb database [32]: SMG1-SMG8-SMG9-UPF1 (pdb id.: 6z3r [33]); SMG5-SMG7 (pdb id.: 3zhe [34]); UPF1-UPF2 (pdb id.: 2wjv [35]); and UPF2-UPF3 (pdb id.: 1uw4 [36]). The central panel represents the cartoon representation of these interactions within the NMD pathway. (**B**) Assembly of genes for the surveillance pathway and the central role in the NMD pathways of the SMG1–UPF2–SMG7 genes identified in our cross-linking MS experiments. After the formation of SURF (SMG1, UPF1, eRF1, and eRF3) within the NMD pathway, this results in the formation of the DECID complex. DECID triggers UPF1 phosphorylation and recruits SMG7 bound with SMG5 [37], and recruitment of SMG7 to UPF1 targets the mRNA containing PTCs for degradation. Interactions of PP2A with SMG7 dephosphorylate UPF1 and prepare a second NMD round. The phosphorylated UPF1 represented as ‘P’ over the NMD machinery diagram.

Comparing the SMG1–UPF2–SMG7 network identified in our cross-linking MS experiments with the existing structures of the SMG1, UPF2, or SMG7 genes highlighted that this network has a crucial role in the degradation of mRNA with PTCs. The following known structures in the rcsb pdb database with different partners [32] are available: SMG1-SMG8-SMG9-UPF1 (pdb id.: 6z3r [33]); SMG5-SMG7 (pdb id.: 3zhe [34]); UPF1-UPF2 (pdb id.: 2wjv [35]); and UPF2-UPF3 (pdb id.: 1uw4 [36]; Figure 4A). Many of the interaction residues from our cross-linking MS have different sites compared to known interactions (Figure 4A), in particular, the UPF1 and UPF3 docks [35,36] with UPF2 where a deficit in cross-linked peptides was traced. The SMG1 binds with SMG8 and SMG9 [23] on the flip side of the conformation when compared to its interactions with UPF2 or SMG7 (Figure 4A). In addition, a similar difference is observed in the interaction pattern of SMG7 with SMG5 [34] and our cross-linked identified partners of the SMG1–UPF2–SMG7 complex (Figure 4A).

The NMD mechanisms have been proposed in previous studies [10,11,37]; herein, based on identified PPIs between SMG1–UPF2–SMG7, we postulated the crucial role of these interactions in the degradation of mRNA transcripts containing PTCs. An aberrant mRNA with PTC at least 50–55 nucleotides upstream of EJC are degraded by a cytoplasmic and translation-dependent NMD process forming complexes such as SURF, DECID, etc. After the formation of SURF (SMG1, UPF1, eRF1, and eRF3) within the NMD pathway, this results in the formation of the DECID complex. DECID triggers the UPF1 phosphorylation and recruits SMG7 bound with SMG5 [37] because the recruitment of SMG7 to UPF1 targets the mRNA containing PTCs for degradation. Moreover, interactions of PP2A with SMG7 dephosphorylate UPF1 and prepare a second NMD round. These suggest the central role in the NMD pathways of the SMG1–UPF2–SMG7 genes identified in our cross-linking MS experiments (Figure 1 and Figure 2).

### 2.2. Effects of Cancer-Derived Mutations on the Identified Protein Cross-Links

**Figure 5:**
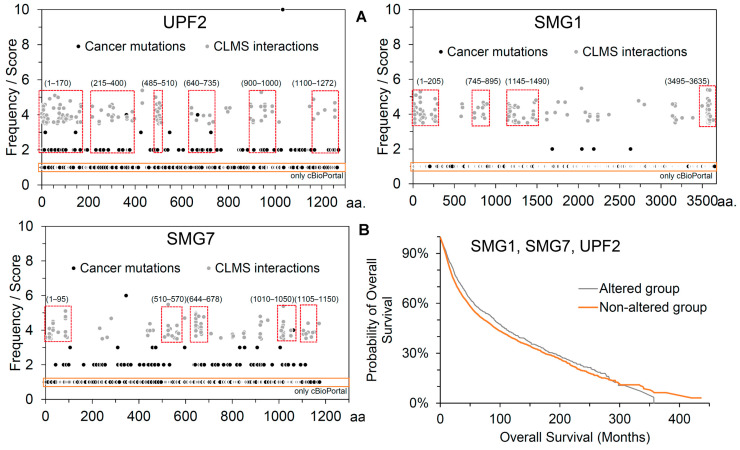
Influence of cancer mutations on the SMG1–UPF2–SMG7 network. (**A**) Cancer-derived mutational landscape compared with the cross-linked MS-identified PPIs between the SMG1, UPF2, and SMG7 genes. Individual variants were derived from different cancer types from the cBioPortal [38] and COSMIC [39] databases. Specific sites of interactions and mutations in different cancer types are marked with a red box. In addition, the mutations emerging specifically from cBioPortal are highlighted in orange box. (**B**) Probability of overall survival of patients compared with their survival in months of the altered and unaltered groups. Data retrieved from the cBioPortal [38] database.

**Figure 6:**
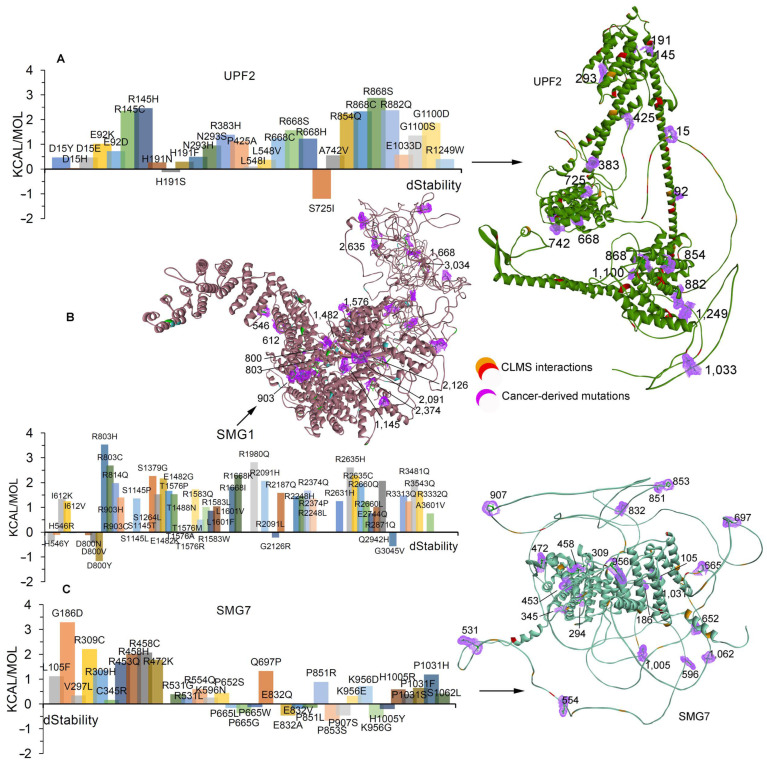
Effect of cancer-derived mutations on structural stability (kcal/mol) of SMG1, UPF2, and SMG7 genes. (**A**–**C**) Change in the structural stability (kcal/mol) of the cross-linked identified UPF2, SMG1, and SMG7, respectively. Mutations with significant effects on the protein structure are highlighted as surfaces in purple colour, and cross-linking interactions are in red/orange colour. High-frequency (≥4) mutations, as well as those common to cBioPortal [38] and COSMIC [39] databases, were investigated further to determine the effects of point mutations on the structure of the SMG1, UPF2, and SMG7 genes. The black line over the protein structures represents the location of a particular amino acid.

Considering the crucial role of the NMD pathway in degrading mRNA containing PTCs and its involvement in different cancer types, we retrieved a dataset of cancer-derived mutational landscapes from cBioPortal [38] and COSMIC [39] databases and compared them with the cross-linked MS-identified PPIs between the SMG1, UPF2, and SMG7 genes (Figure 5). Several sites of PPIs mutated in different cancer types were revealed (Figure 5) when comparing the cross-linked identified proteins and cancer mutations. For the UPF2 protein, several variants retrieved from cBioPortal [38] and COSMIC [39] were found to be interacting with SMG1 or SMG7. In particular, the 1–170, 215–400, 485–510, 640–735, 900–1000, and 1100–1271 amino acids from the UPF2 were found common in the cross-linking MS experiments and cancer-derived mutations. Similarly, the residues within the ranges of 1–205, 745–895, 1145–1490, and 3495–3535 from the SMG1 gene interacting with UPF2 or SMG7, and 1–95, 510–570, 644–678, 1010–1050, and 1105–1150 in SMG7 were mutated in different cancers. The overall patient survival status relating to SMG1, UPF2, and SMG7 suggests that there is a substantial decrease in survival for the patients from the altered group (Figure 5B). To investigate their functional impact on the identified cross-linked network proteins, we computed structural stability upon interesting point mutations (coming from the protein–protein interfaces) of their structures (Figure 6).

Effects of cancer-derived mutations with high-frequency (≥4) mutations as well as those common to cBioPortal [38] and COSMIC [39] databases were studied to identify changes in the structural stability of SMG1, UPF2, and SMG7 structures (Figure 6 and Table S2). Most of the cancer mutations emerge in the interaction sites of UPF2 in our cross-linking MS experiments, in particular, the H191S induced stability (kcal/mol) within the structure whereas H191N/F reduced the stability (Figure 6A). The following mutations were found showing significant effects on the UPF2 structure: S725I induced stability, whereas D15Y/H/E, E92K/D, R145C/H, N293S/H, R383H, P425A, L548I/V, R668C/S/H, A742V, R854Q, R868C/S, R882Q, E1033D, 1100D/S, and R1249W destabilize the structure (Figure 6A). Among the identified mutations with functional effects on the structure, the R145H/C and R868S/C variants significantly destabilized the structure with higher energy (kcal/mol) and are located at the SMG1 binding site of UPF2 (identified in our cross-linking datasets; Figure 6A).

For SMG1, the following mutations induced stability within the structures—H546Y, D800N/V/Y, T1576A, G2126R, and G3034V—whereas any mutations at positions I612K/V, R803H/C, R903C/H, S1145P/T/L, E1482G/K, R1583W/Q/L, L1601F/V, R1668I/K, R2091H/L, R2374P/Q, and R2635C/H had a negative effect on the structural stability (kcal/mol; Figure 6B). The T1576A variant-induced stability and T1576P/M have shown contradictory effects (Figure 6B). Among the studied cancer-derived mutations, R803H, R1980Q, and R2635H highly destabilized the SMG1 structure (Figure 6B). The effect of mutations over different sites of SMG1 revealed that variants in the interface with UPF2 or SMG7, identified in our cross-linking experiment, have shown significant stability effects, whereas in the SMG8 or SMG9 sites, they have very minimal effect (Figure 6B). Moreover, within the SMG7 structure, the following mutations induced stability: P665L/W/G, E832A/V, P851L, P853S, P907S, K956G, and H1005Y. Inserting point mutations R309H/C, R458C/H, F531G/L, and P1031S/F/H in the SMG7 structure reduced stability of the SMG7 structure (kcal/mol); the P851R, K956E/D, and H1005R residues hindered the stability, whereas P851L, K956G, and H1005Y induced the stability of the structure (Figure 6C). Overall, the effects of cancer-derived mutations on the SMG7 structure revealed that in most cases they lack any significant effect on the interaction interface with SMG1 or UPF2 (Figure 6C). Mutations showing significant effects on the protein stability and belonging to PPI interfaces in our CLMS experiments can be experimentally validated to trace their negative or positive impact in their intermolecular interactions.

## 3. Materials and Methods

### 3.1. Identification of Protein–Protein Networks within the NMD Machinery

Intermolecular protein–protein interactions are fundamental to the formation of intricate interaction networks and the assembly of multiprotein complexes that represent the functional workhorses of the cell. Detailed understanding of the structure and dynamics of these multimeric and functional entities is critical towards understanding their biological functions. In our previous study [21]/we cross-linked the Flo-1 treated IFNα14 cancer cells with DSS (disuccinimidyl suberate) and identified crucial PPIs with validating co-immunoprecipitation (Co-IP; Figure 1A). Detailed parameters about sample preparation and processing in mass spectrometry were described in our previous work [21]. CLMS has become a standard tool for the topological analysis of multiprotein complexes and can provide information on protein structures. The SIM-XL [40] computational package was used to detect cross-linkers in the homodimer or heterodimer. SIM-XL can eliminate possibilities by only considering cross-link combinations that contain at least one peptide identified with a dead-end modification and by only searching spectra that exhibit tell-tale ions. During CLMS data interpretation, we restricted our screening to selected genes as we were interested in identifying interactions between components of NMD, EJC, and eRFs. Alongside the identified novel SMG1–UPF2–SMG7 complex, there could be other components involved in these interactions; however, the site-specific non-cleavable interactions of the DSS cross-linker with Lysine or Serine surface residues of a gene could have limited their identification in our experiments.

To identify networks within the NMD components, the following parameters were set in the cross-linking mass spectrometry processed samples: the XL mass shift was set to ~138.00 and modification mass shift was set to ~156.00. Different cross-linked sites such as the KK, KS, and KN-TERM, with no reporter ions, were set to identify PPIs. Dynamic DB reduction XCorr threshold was set to 2.5 and dynamic DB reduction minimum number of peptides was set to 2. The higher-energy C-trap dissociation (HCD) fragmentation method was used along with specifying the search with fully specific Trypsin enzyme. The single isotopic possibilities and peaks-matched cutoff were set to 4 minimum AA residues per chain and intra-link maximum charge. Different intra- and intermolecular interactions within the NMD components were presented in the 2D maps in the SML-XL modules. In addition, these interactions were represented by the modelled protein structures of UPF2, SMG1, and SMG7 using the PyMol (The PyMOL Molecular Graphics System, Version 2.0 Schrödinger, LLC; http://www.pymol.org/pymol (accessed on 1 March 2023) package. The BIOVIA Discovery Studio (Dassault Systèmes, BIOVIA Corp., San Diego, CA, USA) and MOE packages (Molecular Operating Environment, 2022.02 Chemical Computing Group ULC, 910–1010 Sherbrooke St. W., Montreal, Canada, 2024) were used for representing the protein structures and identifying different domains of the genes. Interaction network of the eRFs, NMD, and EJC components, and their involvement in different biological pathways were computed using Cytoscape [41].

### 3.2. Homology Modelling and Mutational Landscape of Cross-Linked Genes

Among the identified cross-linked SMG1, UPF2, and SMG7 proteins, several existing structures can be found in the rcsb pdb database [32]; SMG1-SMG8-SMG9-UPF1 (pdb id.: 6z3r [33]), SMG5-SMG7 (pdb id.: 3zhe [34]), UPF1-UPF2 (pdb id.: 2wjv [35]), and UPF2-UPF3 (pdb id.: 1uw4 [36]). However, a full-length structure of UPF2, SMG1, and SMG7 proteins is still not available, which we built using the homology modelling approach implemented in the swiss-model and alpha-fold tools (Figures S4 and S5) [23,42]. UPF2 and SMG1 gained a well-defined modelled secondary structure retrieved from swiss-model tool [23], whereas the SMG7 structure from alpha-fold [42] has more compact regions (Figures S4 and S5). Even though these proteins’ full-length structures were predicted, they have some disordered regions as described in Figures S4 and S5, and, therefore, further optimization of these structures is required prior to protein–protein docking in silico calculations. Herein, these selected full-length structures of UPF2, SMG1, and SMG7 proteins were used to analyze the cross-linked identified amino acids in the PyMol (The PyMOL Molecular Graphics System, Version 2.0 Schrödinger, LLC; http://www.pymol.org/pymol (accessed on 1 March 2023)) program.

An examination of the mutational landscape of NMD, EJC, and eRFs components from the cBioPortal [38] reveals a broad spectrum of mutations occurring in these proteins in a range of cancer types. A key challenge is to understand which ones, among all these mutations, are likely to have a functional impact. We integrate computational methods that can predict the effects of variations on protein structure stability or interactions and can help to identify functionally important mutations. Cancer-derived mutational landscape compared with the cross-link MS identified PPIs between the SMG1, UPF2, and SMG7 genes. Individual variants were derived from different cancer types from the cBioPortal [38] and COSMIC [39] databases. To identify the change in the structural stability of UPF2, SMG1, and SMG7 genes upon inserting the cancer-derived variants, the residue scan or site-directed mutagenesis methodology implemented in the MOE (Molecular Operating Environment, 2022.02 Chemical Computing Group ULC, 910–1010 Sherbrooke St. W., Montreal, Canada, 2024) package was utilized. This approach particularly substitutes the amino acids of interest with other possible replacements and computes a change in the stability (dstability or ΔStability; kcal/mol) within the structure with respect to the wild-type. The LowModeMD ensemble as a conformational search method (implicit vibrational analysis) was used, which considers the residues farther than 4.5 Å distance (from a residue to be mutated) as fixed and residues within this distance will be searched using a LowModeMD active flexible zone. LowModeMD has the effect of searching for minima along the valleys and troughs on the potential energy surface. To analyze the protein structure and inter- or intramolecular interactions, the MOE (Molecular Operating Environment, 2022.02 Chemical Computing Group ULC, 910–1010 Sherbrooke St. W., Montreal, QC H3A 2R7, Canada, 2024) and BIOVIA Discovery Studio Visualizer program (Dassault Systemes, BIOVIA Corp., San Diego, CA, USA) packages were utilized.

## 4. Conclusions

Nonsense-mediated mRNA decay is an important cellular surveillance mechanism that identifies and degrades aberrant mRNA. To identify novel biology by tracing crucial interactions between the NMD components, the Flo-1 cancer cells were treated with IFNa14 and cross-linked using the chemical DSS cross-linker, which was further processed through mass spectrometry techniques. A set of novel complex networks between UPF2, SMG1, and SMG7 from the NMD machinery were identified. Following the SURF complex, the DECID components within the NMD process trigger the UPF1 phosphorylation along with recruiting the SMG7. Recruitment of SMG7 to UPF1 targets the mRNA containing PTCs for degradation. Along with having a crucial role in the NMD process, the SMG1 genes have been shown distinguishable tasks in the alternative splicing of the tumour-suppressor p53 genes. These suggest the central role in the NMD pathways of the SMG1–UPF2–SMG7 genes identified in our cross-linking MS experiments. Comparing these novel SMG1–UPF2–SMG7 networks with existing structures suggests that binding residues from our cross-linking MS have different sites compared to known interactions. UPF1 and UPF3 dock with UPF2 where it lacks any cross-linked peptides and SMG1 binds with SMG8 and SMG9 on the flip side of the conformation when compared to its interactions with UPF2 or SMG7.

Specifically, the CLMS data revealed that UPF2 has a set of binding sites interacting with SMG1 or SMG7 proteins and plays a role in connecting both genes within the NMD pathway. Different inter- and intramolecular interactions were identified within the SMG1–UPF2–SMG7 complex, and based on the intramolecular interactions within the SMG1, it could be a conformational switch of the SMG1 structure when forming networks with different proteins. The UPF2 N-terminal formed most interactions with SMG7, and a set of residues emerged from the MIF4G-I, II, and III domains docked with SMG1 or SMG7. The SMG1 gene was found to have a higher number of interactions with UPF2, mostly with the initial residues, whereas SMG7 formed very few interactions in this region. The PPIs suggest that the SMG1 C-terminal mainly interacts with the UPF2 N-terminal (10–25 aa), MIF4G-I (160–170 and 300–310 aa), and MIF4G-II (490–510 aa) domains, whereas the SMG1 N-terminal interacts with MIF4G-III (940–1000 aa). The SMG1 gene where initial residues formed interactions with UPF2, later was found to bind with SMG7. From the modelling structures of SMG1, UPF2, and SMG7 genes it was observed that the UPF2 and SMG1 interactions emerged from well-defined secondary structures, whereas SMG7 appeared from the connecting loops.

Datasets of mutations for the NMD components derived from different cancer types suggest that these proteins are heavily mutated in several cancers. To investigate their functional impact over the identified cross-linked network proteins, we computed structural stability upon interesting point mutations over their structure. Most of the cancer mutations emerge in the interaction sites of UPF2 in our cross-linking MS experiments, in particular, the H191S induced stability (kcal/mol) within the structure whereas H191N/F reduced the stability. Comparing the effect of mutations over different sites of SMG1 revealed that variants in the interface with UPF2 or SMG7, identified in our cross-linking experiment, have shown significant stability effects, whereas in the SMG8 or SMG9 sites, they have very minimal effect. Cancer-derived mutations in SMG7 lack any significant effect over the interaction interface with the SMG1 or UPF2 proteins. Although previous studies identified NMD inhibitors, there is still an immense need and a place for new therapeutic strategies by exploring novel biology within the NMD machinery. Blocking the NMD pathway could enhance the production of neoantigens or internal (self-derived) cancer vaccines, which could provide a platform to design potential peptide-based vaccines. Our data highlight interactions between SMG1, UPF2, and SMG7 proteins that can be used for potential therapeutic approaches.

## Data Availability

Data is contained within the article and Supplementary Materials.

## Supplementary Materials

The following supporting information can be downloaded at: https://www.mdpi.com/article/10.3390/ijms25063182/s1.
