# Supplementary material for: In Vitro Cross-Linking MS Reveals SMG1–UPF2–SMG7 Assembly as Molecular Partners within the NMD Surveillance
Source: Int J Mol Sci. 2024 Mar 10;25(6):3182. doi: 10.3390/ijms25063182 (PMC10969982; doi:10.3390/ijms25063182)
Supplement: Supplementary file 1 [file ijms-25-03182-s001.zip › U_Kalathiya-Supp-Mat.pdf]

In vitro cross-linking MS reveals SMG1-UPF2-SMG7 assembly as molecular partners within the NMD surveillance

Monikaben Padariya[1,*], Borivoj Vojtesek[2], Ted Hupp[1,3], Umesh Kalathiya[1,*]

[1]International Centre for Cancer Vaccine Science, University of Gdansk, ul. Kładki 24, 80-822, Gdansk, Poland
[2]*Research Centre for Applied Molecular Oncology, Masaryk Memorial Cancer Institute, Zluty kopec 7, 656 53 Brno, Czech Republic*
[3]*University of Edinburgh, Institute of Genetics and Molecular Medicine, Edinburgh Cancer Research Centre, Edinburgh, Scotland, UK*
*Correspondence: monikaben.padariya@ug.edu.pl (M.P) and umesh.kalathiya@ug.edu.pl (U.K)

Supporting Material

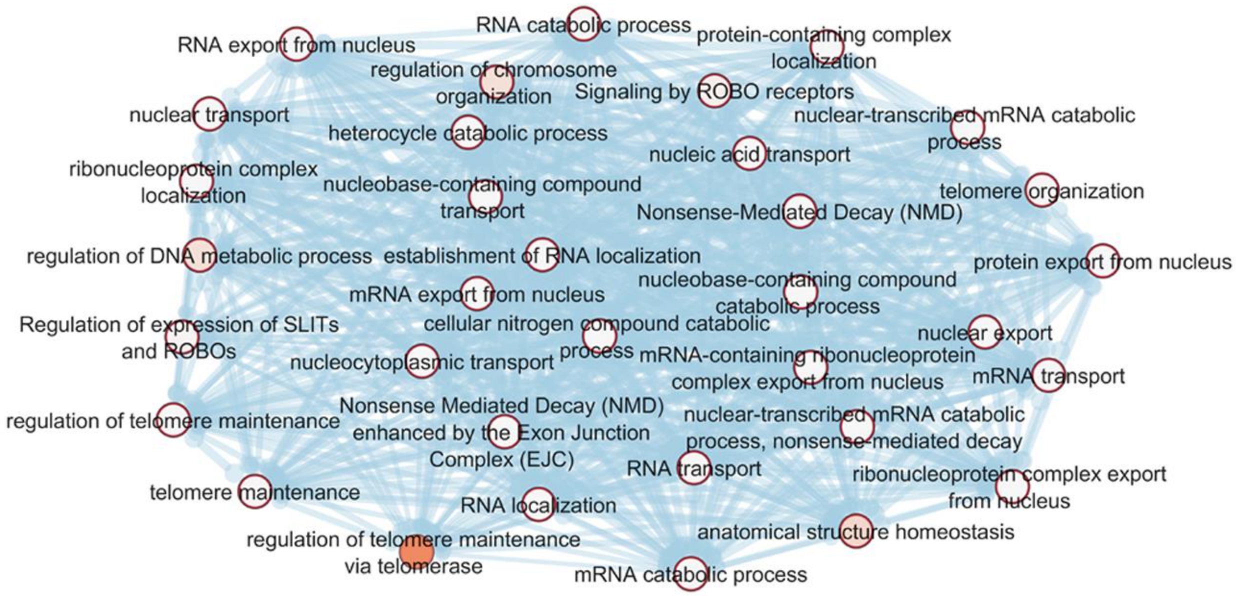

Figure S1. Interaction network of the eRFs, NMD, and EJC components, and their involvement in different biological pathways (computed using Cytoscape). The following NMD components were considered: UPF2, UPF3a, UPF3b, SMG1, SMG5, SMG6, SMG7, SMG8, SMG9, Y14, Magoh, Barent, eIF4AIII, eRF1, and eRF3a.

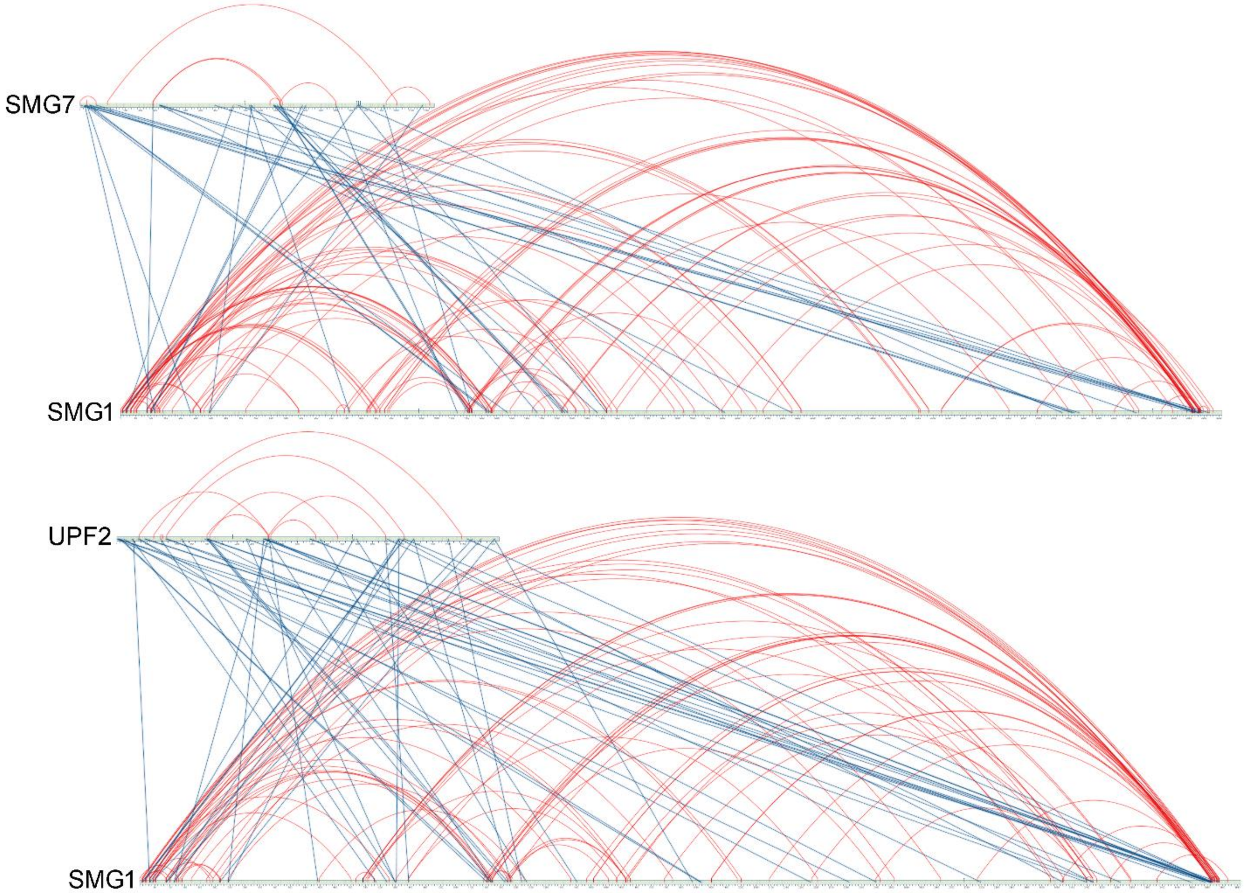

Figure S2. The interactions between SMG1, UPF2, and SMG7 were identified in different biological replicates. Three different independent rounds of sample preparations and analysis were performed as described in our previous work [https://doi.org/10.1038/s41598-022-21393-z]. The Flo-1 cancer cells were treated with DSS cross-linker and IFNa14 interferon to identify upregulated protein-protein interactions (PPIs).

SMG1 (34 % seq coverage)

001 **MSRRAPGSRL SSGGGGGGTK YPRSWNDWQP RTDSASADPD NLKYSSSRDR GGSSSYGLQP** 060
061 **SNSAVVSRQR HDDTRVHADI QNDEKGGYSV NGGSGENTYG RKSLGQELRV NNVTSPEFTS** 120
121 **VQHGSRALAT KDMRKSQERS MSYSDESRLS NLLR**RITRED DRDRRLATVK **QLKEFIQQPE** 180
181 **NK**LVLVKQLD NILAAVHDVL NESSKLLQEL R**QEGACCLGL LCASLSYEAE KIFKWIFSKF** 240
241 **SSSAKDEVK**L LYLCATYK**AL ETVGEKKAFS SVMQLVMTSL QSILENVDTP ELLCKCVK**CI 300
301 LLVARCYPHI FSTNFRDTVD ILVGWHIDHT QKPSLTQQVS GWLQSLEPFW VADLAFSTTL 360
361 LGQFLEDMEA YAEDLSHVAS GESVDEDVPP PSVSLPKLAA LLRVFSTVVR SIGERFSPIR 420
421 GPPITEAYVT DVLYRVMRCV TAANQVFFSE AVLTAANECV GVLLGSLDPS MTIHCDMVIT 480
481 YGLDQLENCQ TCGTDYIISV LNLLTLIVEQ INTKLPSSFV EKLFIPSSKL LFLRYHKEKE 540
541 VVAVAHAVYQ AVLSLKNIPV LETAYK**LILG EMTCALNNLL HSLQLPEACS EIKHEAFKNH** 600
601 **VFNVDNAK**FV VIFDLSALTT IGNAKNSLIG MWALSPTVFA LLSKNLMIVH SDLAVHFPAI 660
661 QYAVLYTLYS HCTR**HDHFIS SSLSSSSPSL FDGAVISTVT TATKK**HFSII LNLLGILLKK 720
721 DNLNQDTRK**L LMTWALEAAV LMKKSETYAP LFSLPSFHKF CKGLLANTLV EDVNICLQAC** 780
781 **SSLHALSSSL PDDLLQR**CVD VCRVQLVHSG TRIRQAFGKL LKSIP**LDVVL SNNNHTEIQE** 840
841 **ISLALRSHMS KAPSNTFHPQ DFSDVISFIL YGNSHRTGKD NWLERLFYSC QRLDKRDQST** 900
901 **IPR**NLLKTDA VLWQWAIWEA AQFTVLSKLR TPLGRAQDTF QTIEGIIRSL AAHTLNPDQD 960
961 VSQWTTADND EGHGNNQLRL VLLLQYLENL EKLMYNAYEG CANALTSPPK VIRTFFYTNR 1020
1021 QTCQDWLTRI RLSIMRVGLL AGQPAVTVRH GFDLLTEMKT TSLSQGNELE VTIMMVVEAL 1080
1081 CELHCPEAIQ GIAVWSSSIV GKNLLWINSV AQQAEGRFEK ASVEYQEHLC AMTGVDCCIS 1140
1141 SFDK**SVLTLA NAGRNSASPK HSLNGESRKT VLSKPTDSSP EVINYLGNK**A CECYISIADW 1200
1201 AAVQEWQNAI HDLK**KSTSST SLNLKADFNY IKSLSSFESG KFVECTEQLE LLPGENINLL** 1260
1261 **AGGSK**EK**IDM KKLLPNMLSP DPR**ELQKSIE VQLLRSSVCL ATALNPIEQD QKWQSITENV 1320
1321 VKYLKQTSRI AIGPLRLSTL TVSQSLPVLS TLQLYCSSAL ENTVSNRLST EDCLIPLFSE 1380
1381 ALRSCKQHDV RPWMQALRYT MYQNQLLEKI KEQTVPIRSH LMELGLTAAK FARKRGNVSL 1440
1441 ATR**LLAQCSE VQLGKTTTAQ DLVQHFKKLS TQGQVDEKWG PELDIEKTKL LYTAGQSTHA** 1500
1501 **MEMLSSCAIS FCK**SVKAEYA VAKSILTLAK WIQAEWKEIS GQLKQVYRAQ HQQNFTGLST 1560
1561 LSKNILTLIE LPSVNTMEEE YPR**IESESTV HIGVGEPDFI LGQLYHLSSV QAPEVAKSWA** 1620
1621 **ALASWAYR**WG RKVVDNASQG EGVRLLPREK SEVQNLLPDT ITEEEKERIY GILGQAVCRP 1680
1681 AGIQDEDITL QITESEDNEE DDMVDVIWR**Q LISSCPWLSE LDESATEGVI K**VWRKVVDRI 1740
1741 FSLYK**LSCSA YFTFLKLNAG QIPLDEDDPR LHLSHR**VEQS TDDMIVMATL RLLRLLVKHA 1800
1801 GELRQYLEHG LETTPTAPWR GIIPQLFSRL NHPEVYVR**QS ICNLLCRVAQ DSPHLILYPA** 1860
1861 **IVGTISLSSE SQASGNK**FST AIP**TLLGNIQ GEELLVSECE GGSPP**ASQDS NKDEPKSGLN 1920
1921 EDQAMMQDCY SKIVDKLSSA NPTMVLQVQM LVAELRRVTV LWDELWLGVL LQQHMYVLRR 1980
1981 **IQQLEDEVKR** VQNNNTLR**KE EKIAIMREK**H TALMKPIVFA LEHVR**SITAA PAETPHEKWF** 2040
2041 **QDNYGDAIEN ALEKLK**TPLN PAKPGSSWIP FK**EIMLSLQQ RAQKRASYIL RLEEISPWLA** 2100
2101 **AMTNTEIALP GEVSAR**DTVT IHSVGGTITI LPTKTKPKKL LFLGSDGKSY PYLFKGLEDL 2160
2161 HLDERIMQFL SIVNTMFATI NRQETPRFHA RHYSVTPLGT RSGLIQWVDG ATPLFGLYKR 2220
2221 WQQR**EAALQA QKAQDSYQTP QNP**GIVPRPS ELYYSKIGPA LKTVGLSLDV SRRDWPLHVM 2280
2281 KAVLEELMEA TPPNLLAKEL WSSCTTPDEW WRVTQSYARS TAVMSMVGYI IGLGDRHLDN 2340
2341 VLIDMTTGEV VHIDYNVCFE KGKSLRVPEK VPFRMTQNIE TALGVTGVEG VFRLSCEQVL 2400
2401 HIMRRGRETL LTLLEAFVYD PLVDWTAGGE AGFAGAVYGG GGQQAESK**QS KREMEREITR** 2460
2461 **SLFSSR**VAEI KVNWFKNRDE MLVVLPKLDG SLDEYLSLQE QLTDVEK**LQG KLLEEIEFLE** 2520
2521 **GAEGVDHPSH TLQHR**YSEHT QLQTQQRAVQ EAIQVKLNEF EQWITHYQAA FNNLEATQLA 2580
2581 SLLQEISTQM DLGPPSYVPA TAFLQNAGQA HLISQCEQLE GEVGALLQQR RSVLRGCLEQ 2640
2641 LHHYATVALQ YPKAIFQKHR **IEQWKTWMEE LICNTTVERC QELYR**KYEMQ YAPQPPPTVC 2700
2701 QFITATEMTL QRYAADINSR LIR**QVERLKQ EAVTVPVCED QLK**EIERCIK VFLHENGEEG 2760
2761 SLSLASVIIS ALCTLTR**RNL MMEGAASSAG EQLVDLTSR**D GAWFLEELCS MSGNVTCLVQ 2820
2821 LLKQCHLVPQ DLDIPNPMEA SETVHLANGV YTSLQELNSN FRQIIFPEAL RCLMKGEYTL 2880
2881 ESMLHELDGL IEQTTDGVPL QTLVESLQAY LRNAAMGLEE ETHAHYIDVA RLLHAQYGEL 2940
2941 IQPRNGSVDE TPKMSAGQML LVAFDGMFAQ VETAFSLLVE KLNKMEIPIA WRKIDIIREA 3000
3001 RSTQVNFFDD DNHRQVLEEI FFLKR**LQTIK EFFRLCGTFS K**TLSGSSSLE DQNTVNGPVQ 3060
3061 IVNVKTLFR**N SCFSEDQMAK PIKAFTADFV R**QLLIGLPNQ ALGLTLCSFI SALGVDIIAQ 3120
3121 VEAKDFGAES KVSVDDLCK**K AVEHNIQIGK FSQLVMNRAT VLASSYDTAW KKHDLVRRLE** 3180
3181 **TSISSCK**TSL QRVQLHIAMF QWQHEDLLIN RP**QAMSVTPP PRSAILTSMK K**KLHTLSQIE 3240
3241 TSIATVQEKL AALESSIEQR LKWAGGANPA LAPVLQDFEA TIAERRNLVL KESQRASQVT 3300
3301 FLCSNIIHFE SLRTRTAEAL NLDAALFELI KRCQQMCSFA SQFNSSVSEL ELRLLQRVDT 3360
3361 GLEHP**IGSSE WLLSAHKQLT QDMSTQR**AIQ TEKEQQIETV CETIQNLVDN IKTVLTGHNR 3420
3421 QLGDVKHLLK AMAKDEEAAL ADGEDVPYEN SVRQFLGEYK SWQDNIQTVL FTLVQAMGQV 3480
3481 RSQEHVEMLQ EITP**TLKELK TQSQSIYNNL VSFASP**LVTD ATNECSSPTS SATYQPSFAA 3540
3541 AVRSNTGQKT QPDVMSQNAR **KLIQKNLATS ADTPPSTVPG TGKSVACSPK KAVRDPKTGK** 3600
3601 AVQERNSYAV SVWKRVK**AKL EGRDVDPNRR MSVAEQVDYV IK**EATNLDNL AQLYEGWTAW 3660

Figure S3. Sequence coverage of SMG1 genes based on the cross-linked peptides identified that are involved in the PPIs. Blue sequences are highlighted showing intermolecular interactions.

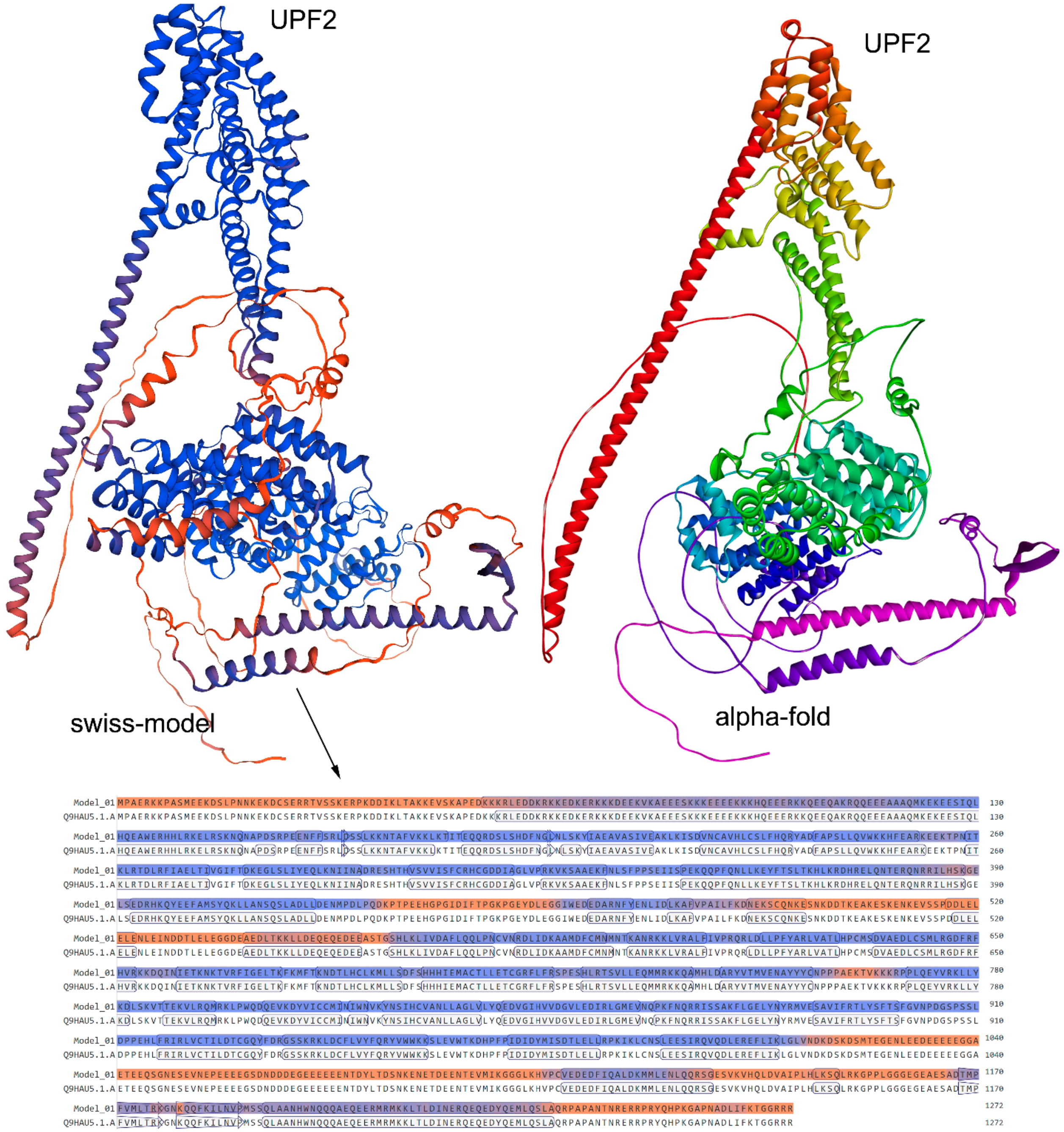

Figure S4. The full-length modelled UPF2 protein structures retrieved from swiss-model [1] and alpha-fold [2]. Below panel represent the secondary structure over the sequence of the modeled UPF2 using the swiss-model tool, the region marked with box represents well-defined structure and the weakly constructed structures are marked in orange. Both tools comparatively generated similar structure.

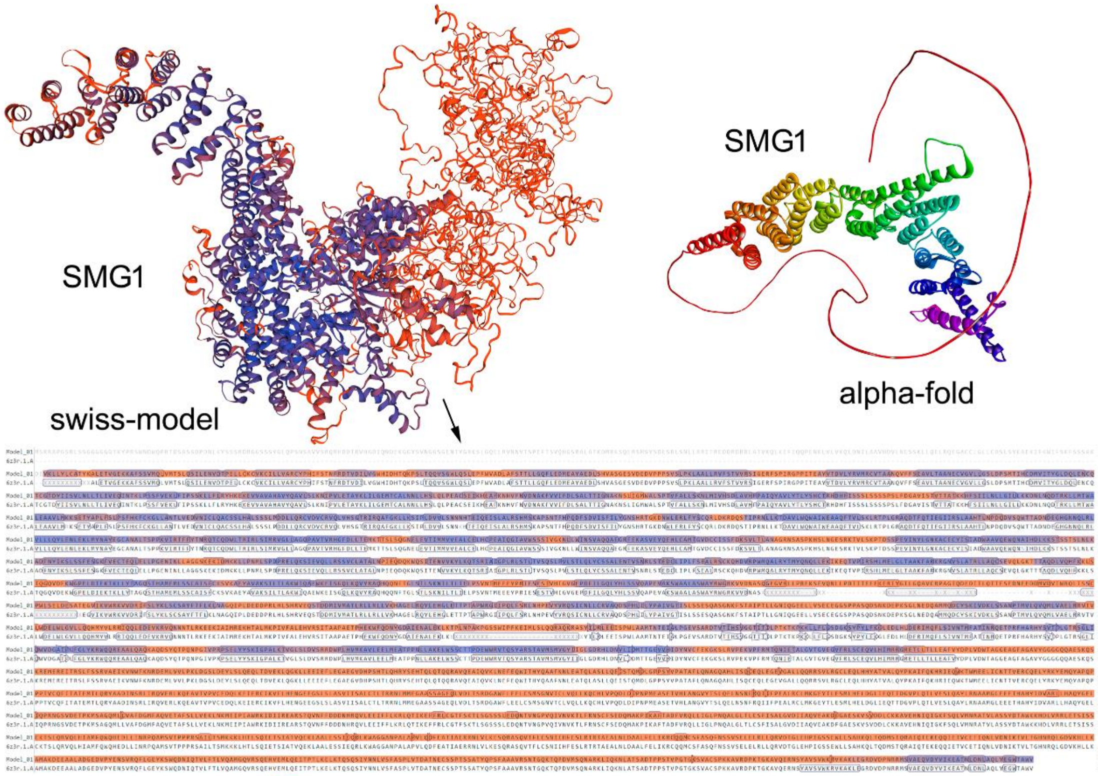

SMG1
swiss-model
SMG1
alpha-fold

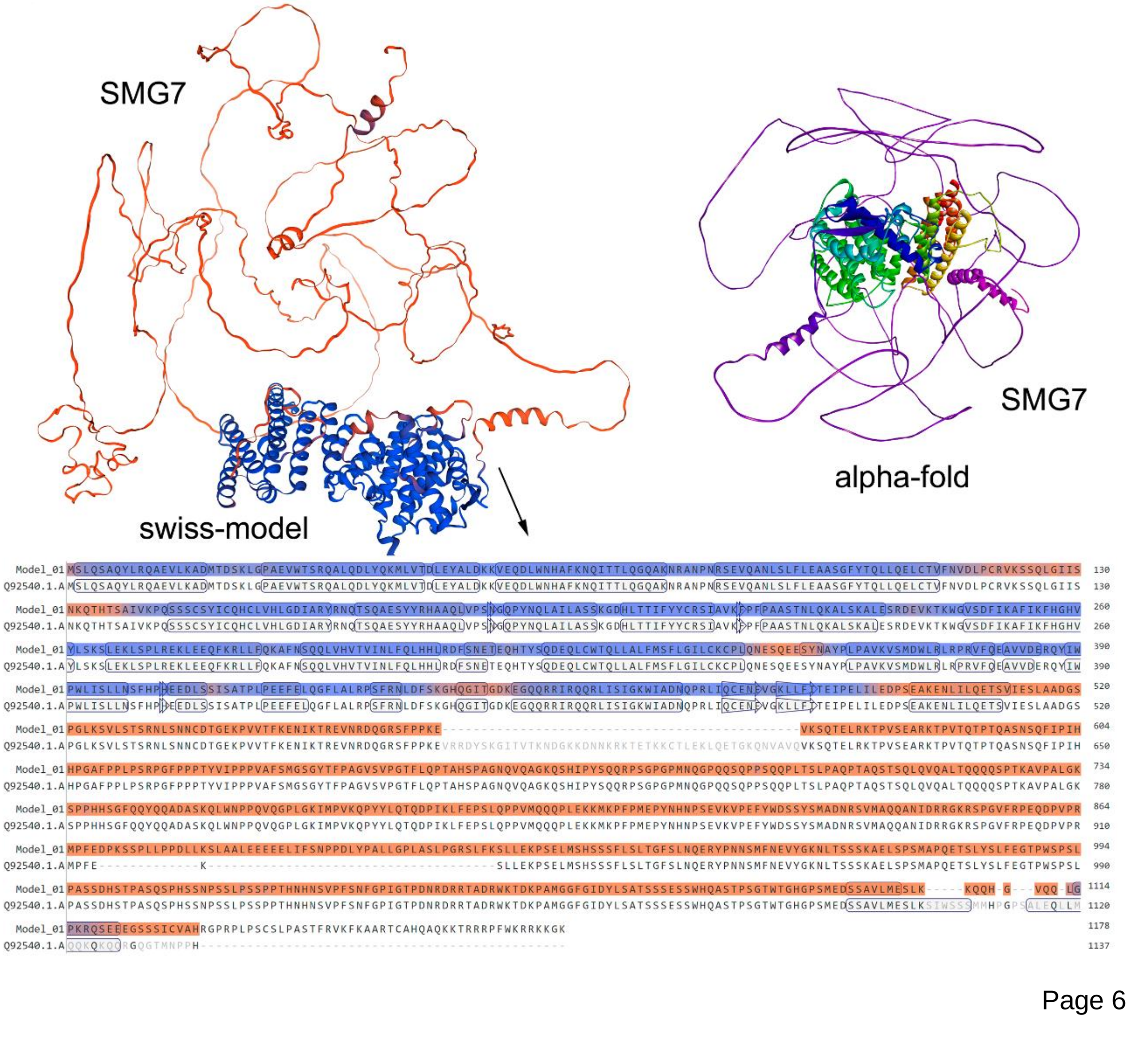

SMG7
swiss-model
SMG7
alpha-fold
Model_01 MSLQSAQYLRQAEVLKADMTDSKLGPAEVWTSRQALQDLYQKMLVTDLEYALDKKVEQDLWNHAFKNQITTLQGQAKNRANPNRSEVQANLSLFLEAASGFYTQLLQELCTVFNVDLPCRVKSSQLGIIS 130
Q92540.1.A MSLQSAQYLRQAEVLKADMTDSKLGPAEVWTSRQALQDLYQKMLVTDLEYALDKKVEQDLWNHAFKNQITTLQGQAKNRANPNRSEVQANLSLFLEAASGFYTQLLQELCTVFNVDLPCRVKSSQLGIIS 130
Model_01 NKQTHTSAIVKPQSSSCSYICQHCLVHLGDIARYRNQTSQAESYYRHAAQLVPSNGQPYNQLAILASSKGDHLTTIFYYCRSIAVKFPFPAASTNLQKALSKALESRDEVKTKWGVSDFIKAFIKFHGHV 260
Q92540.1.A NKQTHTSAIVKPQSSSCSYICQHCLVHLGDIARYRNQTSQAESYYRHAAQLVPSNGQPYNQLAILASSKGDHLTTIFYYCRSIAVKFPFPAASTNLQKALSKALESRDEVKTKWGVSDFIKAFIKFHGHV 260
Model_01 YLSKSLEKLSPLREKLEEQFKRLLFQKAFNSQQLVHVTVINLFQLHHLRDFSNETEQHTYSQDEQLCWTQLLALFMSFLGILCKCPLQNESQEESYNAYPLPAVKVSMDWLRLRPRVFQEAVVDERQYIW 390
Q92540.1.A YLSKSLEKLSPLREKLEEQFKRLLFQKAFNSQQLVHVTVINLFQLHHLRDFSNETEQHTYSQDEQLCWTQLLALFMSFLGILCKCPLQNESQEESYNAYPLPAVKVSMDWLRLRPRVFQEAVVDERQYIW 390
Model_01 PWLISLLNSFHPHEEDLSSISATPLPEEFELQGFLALRPSFRNLDFSKGHQGITGDKEGQQRRIRQQRLISIGKWIADNQPRLIQCENEVGKLLFITEIPELILEDPSEAKENLILQETSVIESLAADGS 520
Q92540.1.A PWLISLLNSFHPHEEDLSSISATPLPEEFELQGFLALRPSFRNLDFSKGHQGITGDKEGQQRRIRQQRLISIGKWIADNQPRLIQCENEVGKLLFITEIPELILEDPSEAKENLILQETSVIESLAADGS 520
Model_01 PGLKSVLSTSRNLSNNCDTGEKPVVTFKENIKTREVNRDQGRSFPPKE--VKSQTELRKTPVSEARKTPVTQTPTQASNSQFIPIH 604
Q92540.1.A PGLKSVLSTSRNLSNNCDTGEKPVVTFKENIKTREVNRDQGRSFPPKEVRRDYSKGITVTKNDGKKDNNKRKTETKKCTLEKLQETGKQNVAVQVKSQTELRKTPVSEARKTPVTQTPTQASNSQFIPIH 650
Model_01 HPGAFPPLPSRPGFPPPTYVIPPPVAFSMGSGYTFPAGVSVPGTFLQPTAHSPAGNQVQAGKQSHIPYSQQRPSGPGPMNQGPQQSQPPSQQPLTSLPAQPTAQSTSQLQVQALTQQQQSPTKAVPALGK 734
Q92540.1.A HPGAFPPLPSRPGFPPPTYVIPPPVAFSMGSGYTFPAGVSVPGTFLQPTAHSPAGNQVQAGKQSHIPYSQQRPSGPGPMNQGPQQSQPPSQQPLTSLPAQPTAQSTSQLQVQALTQQQQSPTKAVPALGK 780
Model_01 SPPHHSGFQQYQQADASKQLWNPPQVQGPLGKIMPVKQPYYLQTQDPIKLFEPSLQPPVMQQQPLEKKMKPFPMEPYNHNPSEVKVPEFYWDSSYSMADNRSVMAQQANIDRRGKRSPGVFRPEQDPVPR 864
Q92540.1.A SPPHHSGFQQYQQADASKQLWNPPQVQGPLGKIMPVKQPYYLQTQDPIKLFEPSLQPPVMQQQPLEKKMKPFPMEPYNHNPSEVKVPEFYWDSSYSMADNRSVMAQQANIDRRGKRSPGVFRPEQDPVPR 910
Model_01 MPFEDPKSSPLLPPDLLKSLAALEEEEELIFSNPPDLYPALLGPLASLPGRSLFKSLLEKPSELMSHSSSFLSLTGFSLNQERYPNNSMFNEVYGKNLTSSSKAELSPSMAPQETSLYSLFEGTPWSPSL 994
Q92540.1.A MPFE-------------K-------------------------------------SLLEKPSELMSHSSSFLSLTGFSLNQERYPNNSMFNEVYGKNLTSSSKAELSPSMAPQETSLYSLFEGTPWSPSL 990
Model_01 PASSDHSTPASQSPHSSNPSSLPSSPPTHNHNSVPFSNFGPIGTPDNRDRRTADRWKTDKPAMGGFGIDYLSATSSSESSWHQASTPSGTWTGHGPSMEDSSAVLMESLK-----KQQH-G---VQQ-LG 1114
Q92540.1.A PASSDHSTPASQSPHSSNPSSLPSSPPTHNHNSVPFSNFGPIGTPDNRDRRTADRWKTDKPAMGGFGIDYLSATSSSESSWHQASTPSGTWTGHGPSMEDSSAVLMESLKSIWSSSMMHPGPSALEQLLM 1120
Model_01 PKRQSEEEGSSSICVAHRGPRPLPSCSLPASTFRVKFKAARTCAHQAQKKTRRRPFWKRRKKGK 1178
Q92540.1.A QQKQKQQRGQGTMNPPH--- 1137

Figure S5. The full-length modelled SMG1 and SMG7 protein structures retrieved from swiss-model [1] and alpha-fold [2]. SMG1 structure constructed using swiss-model have shown well-defined structure whereas, for SMG7 alpha-fold have generate a compacts structure. Below each modeled structure panel represent the secondary structure over the sequence of the modeled SMG1 or SMG7 using the swiss-model tool, the region marked with box represents well-defined structure and the weakly constructed structures are marked in orange.

Supporting Tables attached as separate file:

Table S1. Intermolecular interactions identified using the cross-linking mass spectrometry approach between SMG1, UPF2, and SMG7 from the NMD pathway.

Table S2. Effect of cancer derived mutations on structural stability of SMG1, UPF2, and SMG7 genes. High frequency (≥4) mutations, as well as those common to cBioPortal and COSMIC databases, were investigated further to determine the effect of point mutations on the structure of the SMG1, UPF2, and SMG7 genes.

References

[1] A. Waterhouse, M. Bertoni, S. Bienert, G. Studer, G. Tauriello, R. Gumienny, F.T. Heer, T.A.P. de Beer, C. Rempfer, L. Bordoli, R. Lepore, T. Schwede, SWISS-MODEL: homology modelling of protein structures and complexes, Nucleic Acids Res. 46 (2018) W296–W303. https://doi.org/10.1093/nar/gky427.

[2] Jumper, J.; Evans, R.; Pritzel, A.; Green, T.; Figurnov, M.; Ronneberger, O.; Tunyasuvunakool, K.; Bates, R.; Žídek, A.; Potapenko, A.; et al. Highly Accurate Protein Structure Prediction with AlphaFold. Nature 2021, 596, 583–589.
